# Detection of COVID-19 using edge devices by a light-weight convolutional neural network from chest X-ray images

**DOI:** 10.1186/s12880-023-01155-7

**Published:** 2024-01-02

**Authors:** Sohamkumar Chauhan, Damoder Reddy Edla, Vijayasree Boddu, M Jayanthi Rao, Ramalingaswamy Cheruku, Soumya Ranjan Nayak, Sheshikala Martha, Kamppa Lavanya, Tsedenya Debebe Nigat

**Affiliations:** 1 Department of Computer Science and Engineering, National Institute of Technology Goa, Ponda, 403401 Goa India; 2 Department of Electronics and Communication Engineering, National Institute of Technology Warangal, Hanamkonda, 506004 Telangana India; 3 Department of CSE, Aditya Institute of Technology and Management, Kotturu, Tekkali, Andhra Pradesh India; 4 Department of Computer Science and Engineering, National Institute of Technology Warangal, Hanamkonda, 506004 Telangana India; 5 School of Computer Engineering, KIIT Deemed to be University, Bhubaneswar, 751024 Odisha India; 6 School of Computer Science and Artificial Intelligence, SR University, Warangal, 506004 Telangana India; 7 University College of Sciences, Acharya Nagarjuna Univesity, Guntur, Andhra Pradesh India; 8 Faculty of Computing and Informatics, Jimma Institute of Technology, Jimma, Oromia Ethiopia

**Keywords:** COVID-19, CNN, Deep learning, Chest X-ray images, Light weight model

## Abstract

Deep learning is a highly significant technology in clinical treatment and diagnostics nowadays. Convolutional Neural Network (CNN) is a new idea in deep learning that is being used in the area of computer vision. The COVID-19 detection is the subject of our medical study. Researchers attempted to increase the detection accuracy but at the cost of high model complexity. In this paper, we desire to achieve better accuracy with little training space and time so that this model easily deployed in edge devices. In this paper, a new CNN design is proposed that has three stages: pre-processing, which removes the black padding on the side initially; convolution, which employs filter banks; and feature extraction, which makes use of deep convolutional layers with skip connections. In order to train the model, chest X-ray images are partitioned into three sets: learning(0.7), validation(0.1), and testing(0.2). The models are then evaluated using the test and training data. The LMNet, CoroNet, CVDNet, and Deep GRU-CNN models are the other four models used in the same experiment. The propose model achieved 99.47% & 98.91% accuracy on training and testing respectively. Additionally, it achieved 97.54%, 98.19%, 99.49%, and 97.86% scores for precision, recall, specificity, and f1-score respectively. The proposed model obtained nearly equivalent accuracy and other similar metrics when compared with other models but greatly reduced the model complexity. Moreover, it is found that proposed model is less prone to over fitting as compared to other models.

## Introduction

COVID-19 was initially discovered in late December 2019 in Wuhan, China. Approximately 619.7 million individuals have been impacted by the illness globally to date, and 6.5 million have died as a result (https://covid19.who.int/ ). It is a lung condition brought on by SARS-CoV-2 [1]. Infected individuals are exposed to symptoms like sore throat, headache, loss of taste and smell, cough, chills, and wheezing. Identifying, isolating, and treating the sick while adhering to all preventions is the fundamental strategy for containing this pandemic. The various strains of coronavirus have an ability of causing infections and diseases of high severity such as “Middle East respiratory syndrome” (MERS-CoV) and “Severe Acute Respiratory Syndrome” (SARS-CoV). Due to the type of virus, the World Health Organization (WHO) named the pandemic COVID-19 in February 2020 [2]. This virus is mainly airborne, along with other media such as contaminated surfaces, and acts in various manners based on the immunity of the infected, which can be asymptomatic, benign or even severe [3]. This has caused the health systems of even developed countries such as the United States of America to fail, which has approximately 94 million cumulative cases as of September 19, 2022.

The RT-PCR test is the most frequently used technique for identifying COVID-19. The initial RNA is subsequently broken down, dsDNA is created, and PCR amplification occurs as usual during RT-PCR. However, RT-PCR has some drawbacks. The RT-PCR test is a time-consuming procedure [4]. Patients who have the device placed deep within their noses or mouths must also endure severe treatment [5].

Other methods can be employed, including computed tomography (CT Scan) and Chest X-ray (CXR) imaging. When comparing CT Scan to CXR, CT Scan is time-consuming, expensive, less accurate and subjects patients to greater risks. Chest radiograph, on the other hand, is available in the majority of hospitals and clinics, making it more widely accessible [6]. The rib cage of a person may be seen with CXR. The CXR approach is typically the primary choice if radiologists can identify the chest pathology. Considering the circumstance, the COVID-19 is detected using the CXR technique [7, 8]. As a result, the only focus of this study is on utilising X-ray imaging to identify COVID-19 patients in the future.

The development of automatic detection methods based on artificial intelligence is required due to the rapid expansion of the COVID-19 pandemic. It is challenging to locate skilled physicians for each institution because of the shortage of skilled radiologists in various locations. Additionally, artificial intelligence algorithms will help patients by swiftly, easily, and accurately resolving this problem. The vast training of radiologists in this field is a key aspect. AI can aid in making a precise diagnosis in radiology [9]. Convolutional neural networks, among other deep learning-based AI systems, were employed in conjunction with chest x-rays to get accurate results utilising COVID-19. This is so because CNNs can learn the features on their own without the user having to extract them. Deep CNNs have been used to solve a variety of problems, including classification of skin cancer, arrhythmia diagnosis, brain disease categorisation, breast cancer detection, fundus image segmentation, lung segmentation, and lung detection of pneumonia using X-ray pictures. Deep learning’s performance has allowed Canadian start-up DarwinAI to feel that she has created a tool that could aid doctors in making important choices. As a result, DarwinAI is quite positive about the utilisation of AI in the struggle against COVID-19 [3].

The model we propose is built utilising numerous serial and parallel layers with various kernel sizes to identify local and global attributes and link residues to other layers to share information. It is based on GBRAS-Net [10]. To discriminate COVID-19 instances from normal cases, pneumonia cases, and lung opacities, the model was trained from 14,815 chest X-ray images, validated on 2,116 images, and is tested on 4,233 images of a single data set, which is open source and publicly accessible. The outcomes of the suggested technique are also compared with those of previous studies that have been published in the literature. The contributions are summarized as follows:

- The development of a light and accurate deep CNN classifier for quick identification of COVID-19 in order to aid in early diagnosis.
- Using a chest X-ray, which is cost effective compared to other imaging techniques, to differentiate COVID-19 patients through an experimental examination of our deep model.
- Comparison of our model’s performance to state-of-the-art models.
- Helps researchers carry on the development of AI techniques to curb the COVID-19.

The Related work section provides a comprehensive overview of the research conducted by scholars that is pertinent to the current study. The suggested model is presented directly after the associated related work part, in the form of a section called Proposed Methodology. The Experiments section presents the specifics of the conducted tests, the dataset used, the alternative models that were compared to the current model, and the analysis. The final remarks have been offered in the Conclusion section.

## Related work

Ouchicha et al. developed the CVDNet [3] model. They made use of the Kaggle’s “COVID-19 Radiography Dataset” [11, 12]. A total of 2905 chest x-ray images were included in the dataset, and they were classified into three groups: viral pneumonia, COVID-19 and normal. They employed a 5-fold cross-validation technique and partitioned the dataset into 5 equal sections to test and train their model. Four parts of this dataset are used for training, validation, and testing, with the fifth component being used as a stand-alone component. Results are obtained, and analysis is done based on the component that was evaluated. For three classes, this model averaged 96.72 percent precision, 96.69 percent accuracy, 96.84 percent recall, and 96.68 percent F1-score.

A model called CoroNet [13] was created by Asif et al. They used two sources to create their dataset [14, 15]. They gathered 1,300 photos altogether from both of these sources. The dataset included the classes Normal, COVID-19, Bacterial Pneumonia, and Viral Pneumonia. They changed all of the photos to have a resolution of 72 dpi and a size of 224 * 224 pixels. Instead of a dropout layer with two fully connected layers, the core model, Xception, was used. This dataset was divided into 4 sections using the 4-fold cross-validation method: 3 for training & 1 for validation. For testing, they used a dataset produced by Wang et al. [16] & Ozturk et al. [17].

The model LMNet [18], a lightweight multi-scale CNN architecture, was created by Dwivedy et al. The 6426 CXR pictures from three classes-COVID-19, Normal, and Pneumonia (https://www.kaggle.com/prashant268/chest-xray-covid19-pneumonia) make up the data sample used in this study. The values of each pixel are normalised to range from 0 to 1. This original dataset is split such that 4/5th of the dataset went for training, while 1/5th went for testing. Additionally, they added data to the existing dataset to create variations. Overall accuracy for LMNet was 96.03%, with average values for precision, recall, and F1-score - 0.97, 0.96, and 0.96, respectively.

A CNN model containing a GRU (Gated Recurrent Unit) [19] was created by Shah et al. They obtained the data from Cohen et al. [14] and the Kaggle repository. There were three classes in the dataset: COVID-19, Pneumonia, and Normal. The dataset is divided into three groups: training, validation, and testing, with respective ratios : 70%, 10%, and 20%. The average scores for recall, precision, and f1-score are 0.96, 0.96, and 0.95, respectively.

Amir et al. designed a model named FCOD (Fast COvid Detector) [20]. The dataset they used is from Cohen et al. [14]. The train-test split was 80-20. The model achieved sensitivity, specificity, precision, accuracy and f1-score of 0.93, 0.97, 0.97, 0.96 and 0.96 respectively for binary classification.

Oyelade et al. designed CovFrameNet [21]. The dataset is acquired from 5 sources. These databases are the “COVID-19 X-Ray images” [22], the “National Institutes of Health (NIH) Chest X-Ray Dataset” [14], “COVID-19 Radiography database” [16], “COVIDNet” [11], “COVID-19 Chest X-Ray” [23], and “Actualmed COVID-19 Chest X-Ray Dataset” (https://github.com/agchung/Actualmed-COVID-chestxray-dataset ). All these datasets constitute total 15 classes. The system achieved 1.00, 0.85, 0.85, 0.90, 0.50, and 1.00 for specificity, recall, precision, F-score, AUC, and accuracy.

Castiglione et al. developed ADECO-CNN [24]. The dataset they used was derived from Soares et al. [25]. This approach was compared with VGG19, GoogleNet, and ResNet models based on pre-trained CNNs. Extensive analysis demonstrated that the CNN model optimized for ADECO-CNN could classify CT images with 99.99% accuracy, 99.96% sensitivity, 99.92% accuracy, and 99.97% specificity.

A preliminary literature review on the use of machine learning techniques for COVID-19 detection was conducted by Chiroma et al. [26]. The survey covered different deep learning techniques, including CNN variations like GoogleNet, VGG, Inception, SqueezeNet, Xception, Alexnet, ResNet, MobileNet, etc. It also noted difficulties and made recommendations for further research.

Similarly, Tayarani et al. [27] provided an overview of the numerous applications of Artificial Intelligence (AI) in the fight against COVID-19. To diagnose various symptoms and tests, determine a patient’s COVID-19 severity, conduct image testing, and study epidemiology, artificial intelligence techniques have been utilised. The article covered CNN’s use of CT and X-rays to detect COVID-19. Alimadadi et al. [28] discussed an overview of AI intelligence applications in the fight against the COVID-19 pandemic. Wynants et al. [29] discussed diagnosis and treatment of COVID-19 for its rapid recognition based on several models, including machine learning.

## Preliminaries

### Formalisation

With a dataset of NS training samples $D=\{X,Y_{0}\}$ provided, we investigate the supervised learning problem. Let $X=\{x_{1},\ldots ,x_{NS}\}$, and $Y_{0}=\{y_{1},\ldots ,y_{NS}\}$ be the sets of associated ground truth labels and input chest x-ray data, respectively, and let $x_{i} \in [{0},{1},\ldots ,{L-1}]^{H*W}$ represent height, width, and the highest greyscale value, respectively, and let $y_{i} \in [0, 1]$ represent Non-COVID and COVID, respectively. Our classifier is expressed as the function $f_{w}: X \rightarrow Y$. The weight parameter in this case is *w*. In the event that the output space Y differs from the label space, the final prediction is obtained using the function $g: Y \rightarrow Y_{0}$. We want to minimize the training set’s prediction error rate, which measures how different $f_{w}(x)$ is from its ground truth category. In order to identify values for *w* that will lower the error function on the training sample, we are iteratively executing the training procedure.

### Convolutional neural network

We were encouraged by the work done on ResNet [30]. In ResNet, we need not connect every layer in a sequence. We can connect some layers far behind in the model to layers far ahead. This means that a layer receives input not only from its immediate previous layer but from also other layers behind. So a layer can be a function of multiple previous layers, and many other layers can be a function of one or more layers. This is achieved by using skip-connections. The end layer receives both the non-processed/partially processed input and fully processed input *F*(*x*). Both the results are added $x:H(x) = F (x) + x$. The actual work done is from GBRAS-Net [10], which we have modified for better results and performance.

Our proposed model CNN possesses two such skip connections. Other layers include 2D Convolution, Depthwise 2D Convolution, Batch Normalisation, 2D Average Pooling, Global 2D Average Pooling and a Dense layer.

#### Convolution layer

The primary component of any CNN is the convolution layer, hence its name. This layer possesses kernels, which are nothing but a square matrix, in most cases, whose values are learned by the model. This layer uses a convolution operation, which is basically different from matrix multiplication. The operation is formalised as follows:

$$\begin{aligned} F(i, j) = (I*K)(i, j) = \Sigma _{m}\Sigma _{n} I(i+m, j+n) K(m, n) \end{aligned}$$

I = image, K = 2D filter and F = feature map. Dimension of K is m*n.

There are some features which are alike in the entire dataset, or at least in its major portion. These features are locally present in images which have a great role in classifying the image. These features are detected by this layer and the output formed as a result of this calculation is the feature map. Each convolutional layer’s output is fed into an activation function, which creates nonlinearity.

#### Depthwise convolution layer

In case of a multi-channel image,a convolution layer works as follows: A single convolution operation gets performed on all the channels. Consider an image of dimension 3*256*256 and filter 3*3*3, the entire cube filter gets applied on the image cuboid. In depthwise convolution, our image is split into 3 2D matrices of dimensions 256*256, along with the filter turning into 3 matrices of dimensions 3*3. So first image is convoluted with first filter, second image with the second filter and third image with the third filter. Once we obtain all the three results, they are stacked back to a 3D feature map (https://medium.com/@zurister/depth-wise-convolution-and-depth-wise-separable-convolution-37346565d4ec).

#### Activation functions

Because convolution and pooling are all linear procedures, they mix when additional layers of convolution or pooling are applied. This makes any attempt to bring depth to our CNN a futile one. Following each convolution operation, the convolutional neural network modifies the convoluted function to add nonlinearity to the model. This article examines three activation functions:

- **ELU** The ELU (Exponential Linear Unit) is formalised as follows:
  $$\begin{aligned} {ELU(x)} = \left\{ \begin{array}{ll} x,&{}{\text {if}}\ x\ge 0,\\ {\alpha *(e^x-1),}&{}{\text {otherwise.}} \end{array}\right. \end{aligned}$$
  The value at which an ELU saturates for negative net inputs is controlled by the $\alpha$ hyperparameter. The vanishing gradient effect is eliminated with ELUs. The average activations are pushed closer to zero since ELUs have negative values. Because they approach the gradient to the natural gradient, mean activations that are nearer to zero enable learning to occur more quickly. When the argument is decreased, ELU reaches a saturation point and turns negative. A modest derivative is referred to as saturation when it lowers the fluctuation and information that propagates to the next layer (https://www.tensorflow.org/api_docs/python/tf/keras/activations/elu).
- **3Tanh** The 3Tanh activation function is formalised as follows:
  $$\begin{aligned} {3Tanh(x)} = -3 \frac{1 - e^{2x}}{1 + e^{2x}} \end{aligned}$$
  This returns a value between -3 and 3. As per the experiment conducted by Reinal et al. [10], this acts as the best activation function for the experiment.
- **Softmax** Given the input matrix, the softmax function calculates the class probabilities. The formalisation of softmax function is as follows [3]:
  $$\begin{aligned} softmax (z)_j = \frac{e^{z_j}}{\sum _{k=1}^{K} e^{z_k}} \end{aligned}$$
  where $z = [z_1, z_2,$...$, z_K]$ is the input vector to the softmax function, the output of $softmax(z)_{j}$ spans between 0 and 1 and
  $$\begin{aligned} \sum _{j=1}^{K} softmax(z)_{j} = 1 \end{aligned}$$

#### Batch normalisation

This method called batch normalisation (BN) greatly enhances convergence during training. It entails averaging and normalising the network layer output variance [3]. We are given a mini-batch B = {$x_{1},x_{2},\ldots ,x_{m}$} of size m, the normalised values $\hat{x_{1}}, \hat{x_{2}}, \ldots , \hat{x_{m}}$ and corresponding linear transformations $y_{1},y_{2},\ldots ,y_{m}$. The transformation $BN_{\gamma ,\beta }$: $x_{1},x_{2},\ldots ,x_{m} \rightarrow y_{1},y_{2},\ldots ,y_{m}$ is referred to as batch normalisation and is calculated as

$$\begin{aligned} \mu _{B} = \frac{1}{m} \sum _{i=1}^{m} x_{i} \end{aligned}$$

$$\begin{aligned} \sigma ^{2}_B = \frac{1}{m} \sum _{i=1}^{m} (x_{i}-\mu _{B}) \end{aligned}$$

$$\begin{aligned} \hat{x_i} = \frac{x_{i}-\mu _{B}}{\sqrt{\sigma ^{2}_B + \epsilon }} \end{aligned}$$

$$\begin{aligned} y_i = \beta + \gamma \hat{x_i} \equiv BN_{\gamma ,\beta } (x_i) \end{aligned}$$

#### Pooling layer

The characteristics of the convolution layers are gradually decreased in size while still retaining the most crucial data in the pooling layer. This layer lowers the calculations and the number of variables in the network. A window of size $h_p$ * $h_p$ that travels in step $s_p$ on every feature map defines the pooling operation. It is frequently tackled using two basic strategies [3]:

- Max-pooling: This technique involves returning the highest local value possible for each grouping window.
- Avg-pooling: Computes the mean of the local data for each grouping window.

#### Global pooling layer

The Flatten layers are replaced in CNN by the global pooling layer. It creates one feature map for each associated classification task category in the final convolution layer. The mean/maximum of every feature map is computed, and the resultant vector is placed straight to the softmax layer, as opposed to stacking fully linked layers on top of the feature maps. The benefit of global average pooling is that it implements associations among both feature maps and categories, which makes it more intuitive to the convolutional structure. Since there are no variables to optimise with global average/max pooling, overfitting is avoided at this layer. Spatial data is summarised using global average/max pooling in an effort to make it more resilient towards input spatial translations. Global average/max pooling may be thought of as a structural preprocessing step that formally mandates that feature maps be concept (category) accuracy maps (https://androidkt.com/explain-pooling-layers-max-pooling-average-pooling-global-average-pooling-and-global-max-pooling/).

#### Fully connected layer

Every neuron in the area corresponding to this layer’s input, or every neuron in the layer preceding, is linked to every neuron in that layer. This layer generates a vector with K dimensions, where K is the number of categories that the network can predict. This vector holds the probability of categorisation for each image class. A FC layer defines the connection between the classes and the image. Since the record represents the result of the previous layer, it corresponds to the object’s map: high value represents the object’s location in the image.

## Proposed methodology

**Fig. 1:**
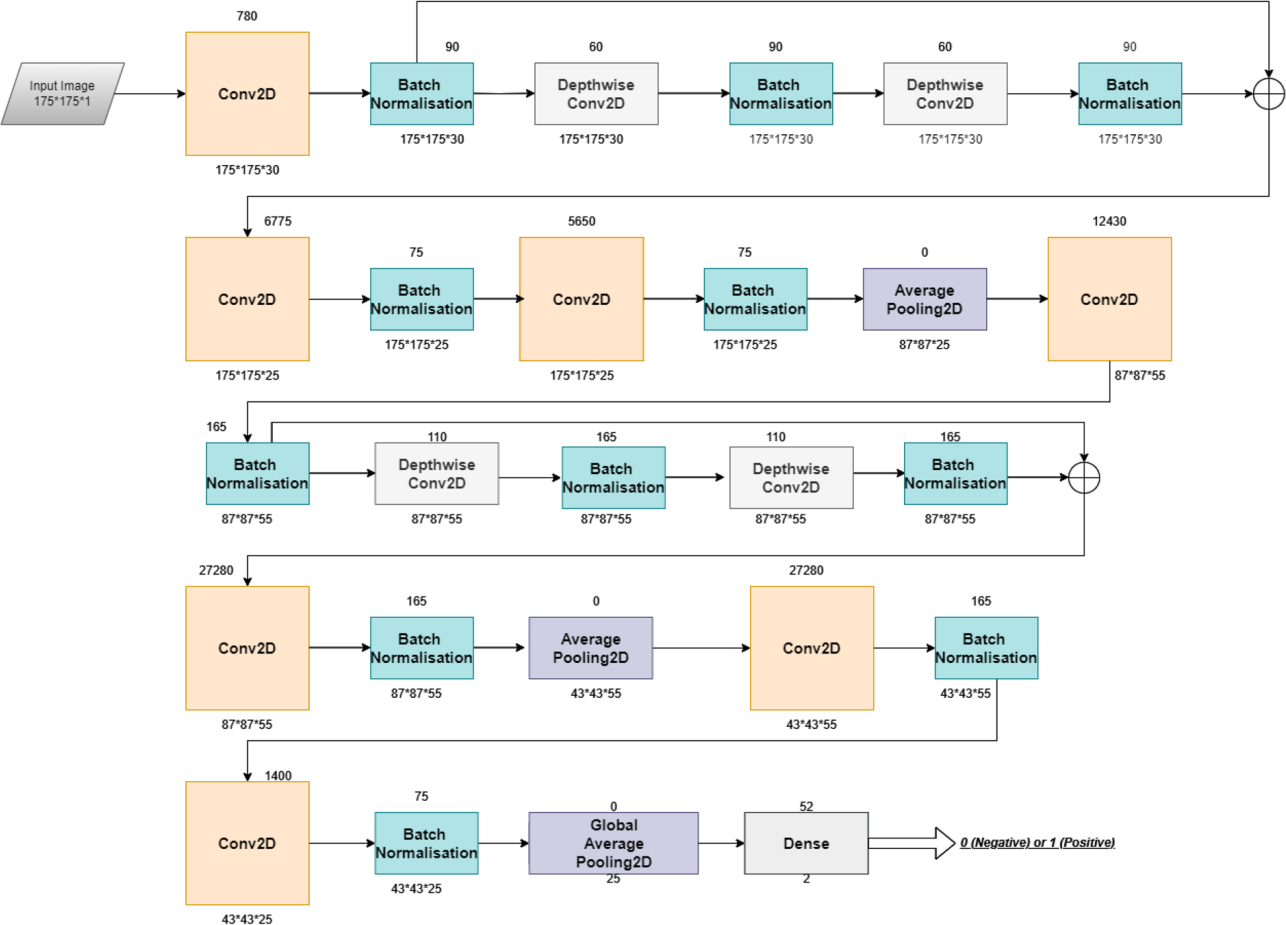
Proposed Architecture

To simplify the explanation, each set of layers is presented in a section of its own. The propped model is given in Fig. 1 and layer wise hyper parameters are summarized in Table 1

**Table 1:** Layered architecture of the proposed model

Layer	Number of filters	Kernel Size	Strides	Output Shape	Number of parameters	Connected to
Input				175*175*1	0	–
2D Convolution layer	30	5*5	1*1	175*175*30	780	Input
Batch Normalisation				175*175*30	90	2D Convolution layer
Depthwise 2D Convolution layer		1*1	1*1	175*175*30	60	Batch Normalisation
Batch Normalisation 1				175*175*30	90	Depthwise 2D Convolution layer
Depthwise 2D Convolution layer 1		1*1	1*1	175*175*30	60	Batch Normalisation 1
Batch Normalisation 2				175*175*30	90	Depthwise 2D Convolution layer 1
Add				175*175*30	0	Batch Normalisation 1
						Batch Normalisation 2
2D Convolution layer 1	25	3*3	1*1	175*175*25	6775	Add
Batch Normalisation 3				175*175*25	75	2D Convolution layer 1
2D Convolution layer 2	25	3*3	1*1	175*175*25	5650	Batch Normalisation 3
Batch Normalisation 4				175*175*25	75	2D Convolution layer 2
2D Average Pooling		2*2	2*2	87*87*25	0	Batch Normalisation 4
2D Convolution layer 3	55	3*3	1*1	87*87*25	12430	2D Average Pooling
Batch Normalisation 5				87*87*25	165	2D Convolution layer 3
Depthwise 2D Convolution layer 2		1*1	1*1	87*87*25	110	Batch Normalisation 5
Batch Normalisation 6				87*87*25	165	Depthwise 2D Convolution layer 2
Depthwise 2D Convolution layer 3		1*1	1*1	87*87*25	110	Batch Normalisation 6
Batch Normalisation 7				87*87*25	165	Depthwise 2D Convolution layer 3
Add 1				87*87*25	0	Batch Normalisation 5
						Batch Normalisation 7
2D Convolution layer 4	55	3*3	1*1	87*87*25	27280	Add 1
Batch Normalisation 8				87*87*25	165	Batch Normalisation 5
2D Average Pooling 1		2*2	2*2	87*87*25	0	Batch Normalisation 8
2D Convolution layer 5	55	3*3	1*1	43*43*25	27280	2D Average Pooling 1
Batch Normalisation 9				43*43*25	165	2D Convolution layer 5
2D Convolution layer 6	25	1*1	1*1	43*43*25	1400	Batch Normalisation 9
Batch Normalisation 10				43*43*25	75	2D Convolution layer 6
2D Global Average Pooling				25	0	Batch Normalisation 10
Dense	2			2	52	2D Global Average Pooling

### Layer 1

As mentioned about the filters in the previous section, we also have an array of 30 elements. All of them are initialised to 1. The 30 filters serve as the weights and the 30 ones serve as the bias for the first 2D convolution layer. Other features of this convolution layer is mentioned in the preprocessing stage. This creates 780 parameters with an output size of 175*175*30. This is followed by batch normalisation. The input is the output of the latest convolution layer. The parameters of the batch normalisation layer is as follows: momentum is 0.2, epsilon is 0.001, center is set to True, scale is set to False, trainable True, fused None, renorm False, renorm clipping None, renorm momentum 0.4, adjustment None. These parameters are same for all batch normalisation layers used in this model. This creates 90 parameters with an output shape of 175*175*30.

### Layer 2

The next layer is 2D depthwise convolution. This receives the output of the latest batch normalisation layer as its input. This layer uses kernel size of 1*1 with stride of 1. In this case too, these parameters remain the same for all 2D depthwise convolution layer. This creates 60 parameters with an output shape of 175*175*30. This is followed by batch normalisation. The input is the output of the latest 2D depthwise convolution layer. Number of parameters and output shape remains the same as layer 1.

### Layer 3

The next two layers are same as the previous two layers: 2D depthwise convolution followed by batch normalisation. All configurations remain the same as layer 2. Then the outputs from the last two batch normalisation layers are added.

### Layer 4

The next layer is the 2D convolution layer. It has 25 kernels of kernel size 3*3 and stride of 1. Other parameters are as follows: activation elu, padding same, kernel initializer glorot uniform. It creates 6775 parameters and its output shape is 175*175*25. This is followed by batch normalisation which creates 75 parameters and its output shape is 175*175*25.

### Layer 5

Layer 5 is same as layer 4. The only difference is that the 2D convolution layer creates 5650 parameters.

### Layer 6

This layer consists of the 2D average pooling layer. This average pooling layer has a kernel size and stride of 2*2. This layer generates no parameters and has an output size of 87*87*25.

### Layer 7

The structure of layer 7 is same as that of layer 5. The difference in the structure is that the convolution layer has 55 kernels. The convolution layer creates 12430 and has an output shape of 87*87*55. The batch normalisation layer has the same output size creating 165 parameters.

### Layer 8

Layer 8 is same as layers 2 and 3. The output shape is 87*87*55. Number of parameters for depthwise convolution is 110, while it is 165 for batch normalisation layer.

### Layer 9

Layer 9 is exactly the same as layer 8. The outputs of layer 7 and 9 are added.

### Layer 10

The structure of layer 10 is same as layer 7. The output size is 87*87*55. Number of parameters for convolution layer is 27280, while it is 165 for batch normalisation.

### Layer 11

This layer is also a 2D average pooling layer, which is same as layer 6. The output size of this layer is 43*43*55.

### Layer 12

The structure of layer 12 is the same as that of layer 7 and layer 10. The number of parameters created are same as layer 10, but the output shape has reduced to 43*43*55.

### Layer 13

Layer 13 has a 2D convolution layer followed by batch normalisation layer. The 2D convolution layer has 25 filters with kernel size of 1, with other parameters same as its previous counterparts. The convolution layer creates 1400 parameters, while the batch normalisation layer creates 75 parameters, both having the output shape of 43*43*25.

### Layer 14

This is the last layer of the model. This model starts with the 2D Global Average Pooling layer. This transforms the input from 43*43*25 to 25. This input then goes to the dense layer, which is the final classifier. This has 2 neurons - one for positive(1) while other for negative(0), using the softmax activation function. Global pooling layer creates no parameters while the dense layer creates 52 parameters.

### Total parameters

This model creates a total of 83,307 parameters, out of which 81,647 are trainable parameters while 1660 are non-trainable parameters.

## Experiments

This section discusses about the experiments that are performed along with the setup used for experiments.

### Experimental Setup

All the experiments are performed on a 64-bit Ubuntu 18.04 operating system. The GPU system’s configuration included 32 GB of RAM, a 16 GB NVIDIA P5000/PCIe/SSE2 GPU, and a 1 TB hard disk with 200 SSD. The GPU machine was equipped with Anaconda 3.7 and PyTorch version 1.10.0, which utilized CUDA 10.2 and NVIDIA Driver 470. All the programs are implemented using PyTorch library.

### Dataset

A research team with members from “Qatar University, Doha, Qatar” and members from “University of Dhaka, Bangladesh” and other members from nations like Pakistan and Malaysia are joined and worked together with the doctors. Initially, they released CXR images for 3 classes - COVID (219), normal (1341) and viral pneumonia (1345). Next its updated with a total of 1200 images of class COVID and added a new class - Lung Opacity (6012) with Normal (10192), COVID (3616) and Viral Pneumonia (1345). These images are of 299*299 resolution in PNG format [11, 12].

The images for COVID class are collected from the following sources:

- 2473 images from Padchest dataset (https://bimcv.cipf.es/bimcv-projects/bimcv-covid19/#1590858128006-9e640421-6711)
- 183 images from a Germany medical school (https://github.com/ml-workgroup/covid-19-image-repository/tree/master/png)
- 559 image from SIRM, Github, Kaggle and Tweeter (https://sirm.org/category/senza-categoria/covid-19/, https://eurorad.org, https://github.com/ieee8023/covid-chestxray-dataset, https://figshare.com/articles/COVID-19_Chest_X-Ray_Image_Repository/12580328)
- 400 images from Github source (https://github.com/armiro/COVID-CXNet)

A total of 10192 normal data is collected in that 8851 from Radiological Society of North America (RSNA) and 1341 from Kaggle (https://www.kaggle.com/paultimothymooney/chest-xray-pneumonia).

**Fig. 2:**
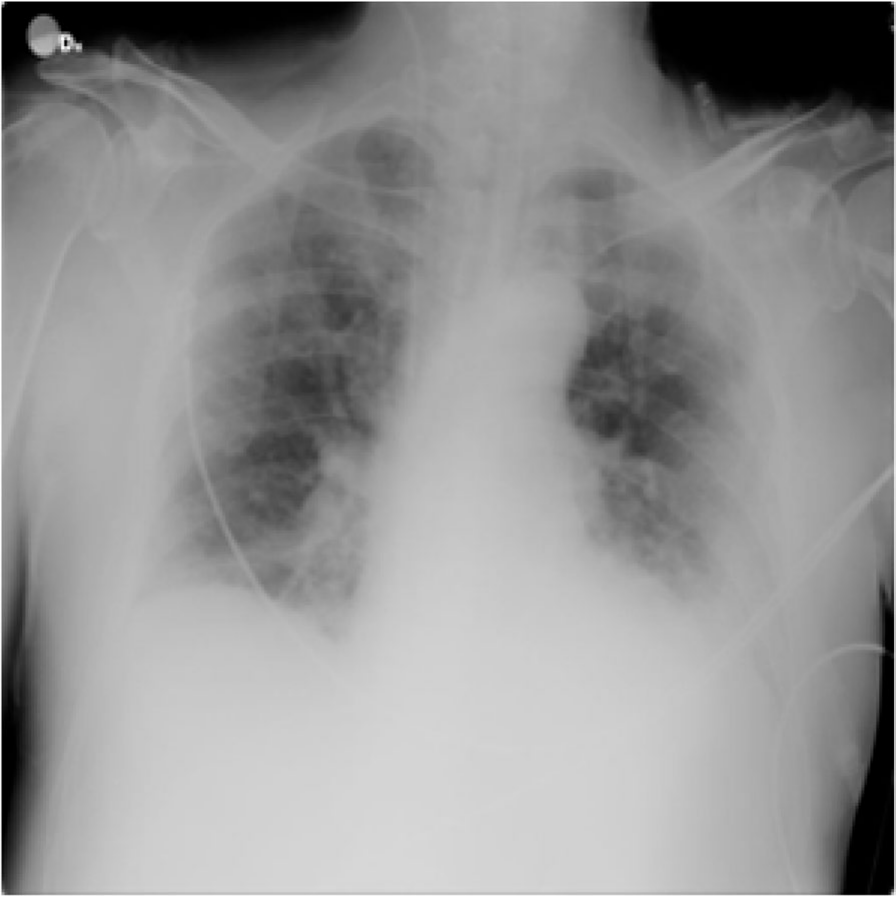
Image with class COVID

**Fig. 3:**
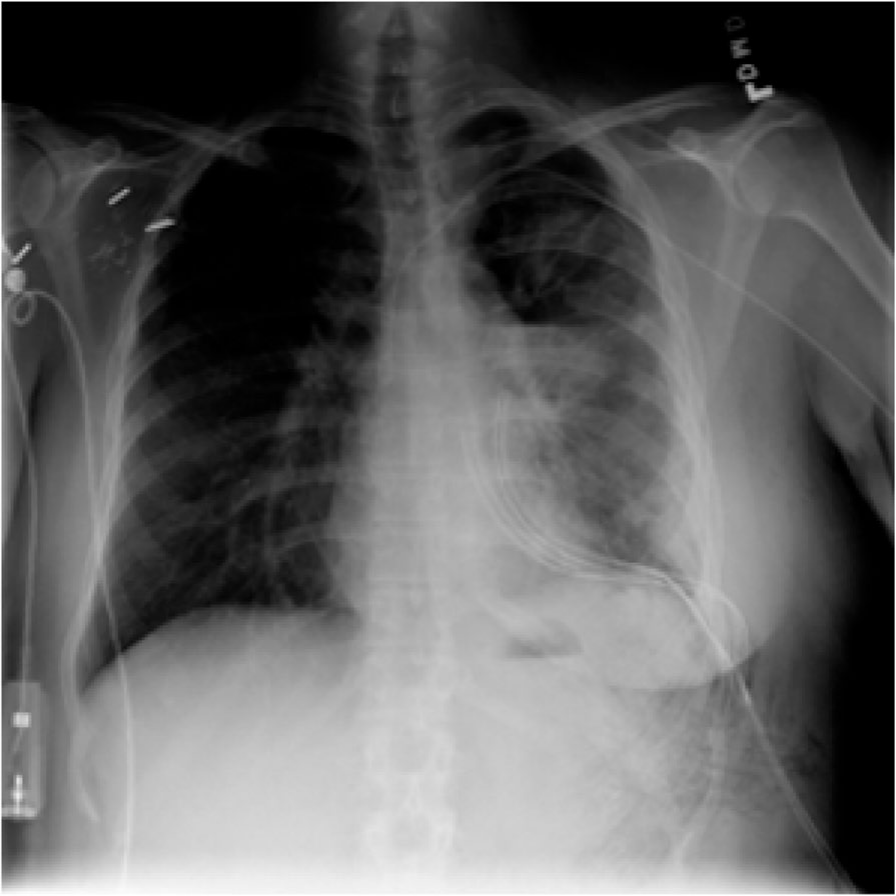
Image with class Lung Opacity

**Fig. 4:**
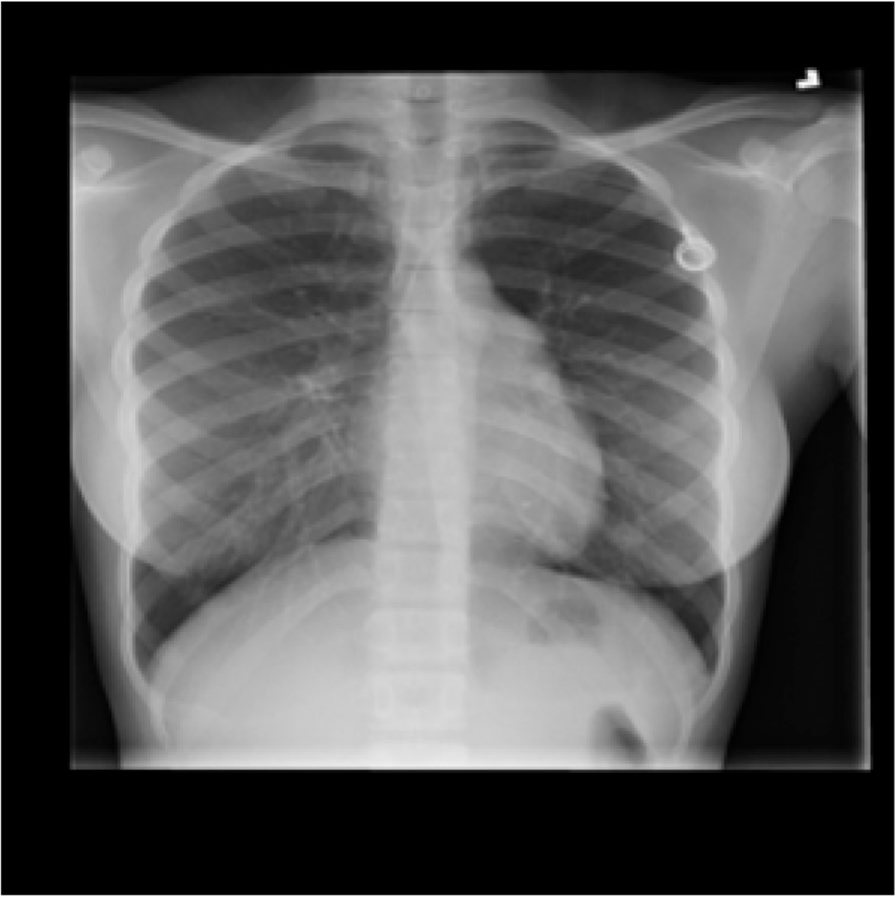
Image with class Normal

**Fig. 5:**
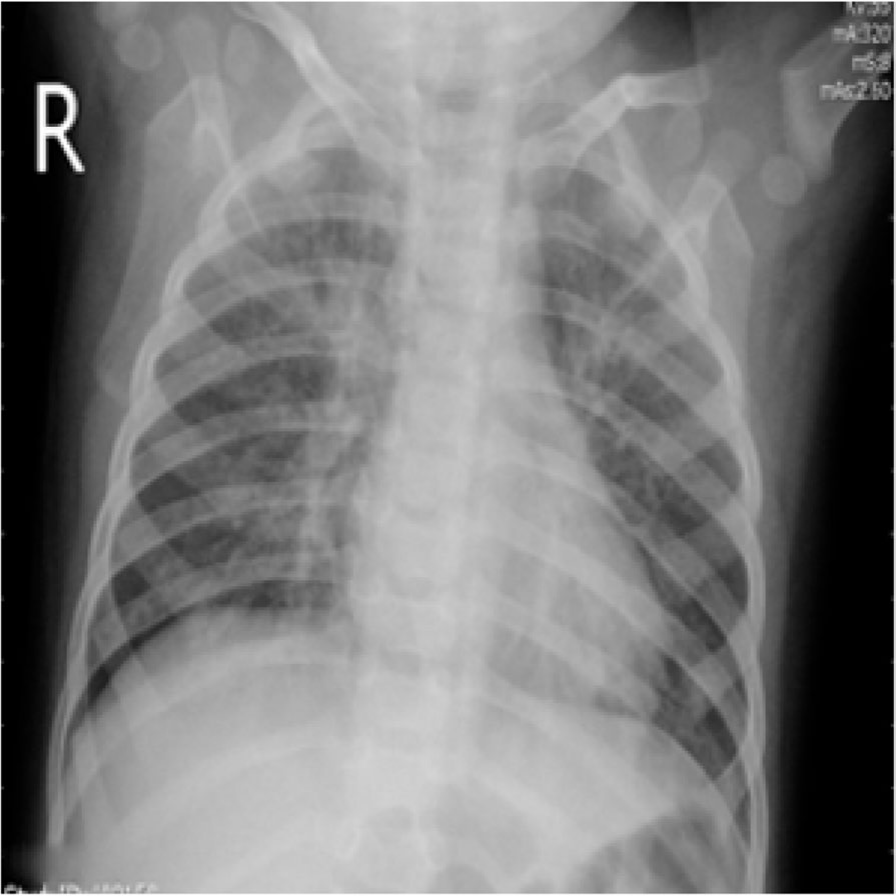
Image with class Viral Pneumonia

For lung opacity, 6012 CXR images are collected from RSNA CXR dataset (https://www.kaggle.com/c/rsna-pneumonia-detection-challenge/data), while for pneumonia, 1345 images are collected from the Chest X-Ray Images (pneumonia) database (https://www.kaggle.com/paultimothymooney/chest-xray-pneumonia). The sample images are shown in Figs. 2, 3, 4 and 5.

### Pre-processing

Most of the images had a black background at the side that require more space in the image. So the black background on all the four sides are removed. Next, each image is resized to 175*175 dimensions. For the class label the ones with COVID positive is marked as 1 while the remaining are marked as negative (0).

After that, the model goes through a pre-processing phase. During this stage a convolution with 30 filters with a size (5, 5) is remained constant throughout the training stage (ie, the layer is not trainable). Next, the convolutional layers are formatted as follows: padding, strides (1, 1), 30 filters (described below), and 3TanH activation function [10].

**Fig. 6:**
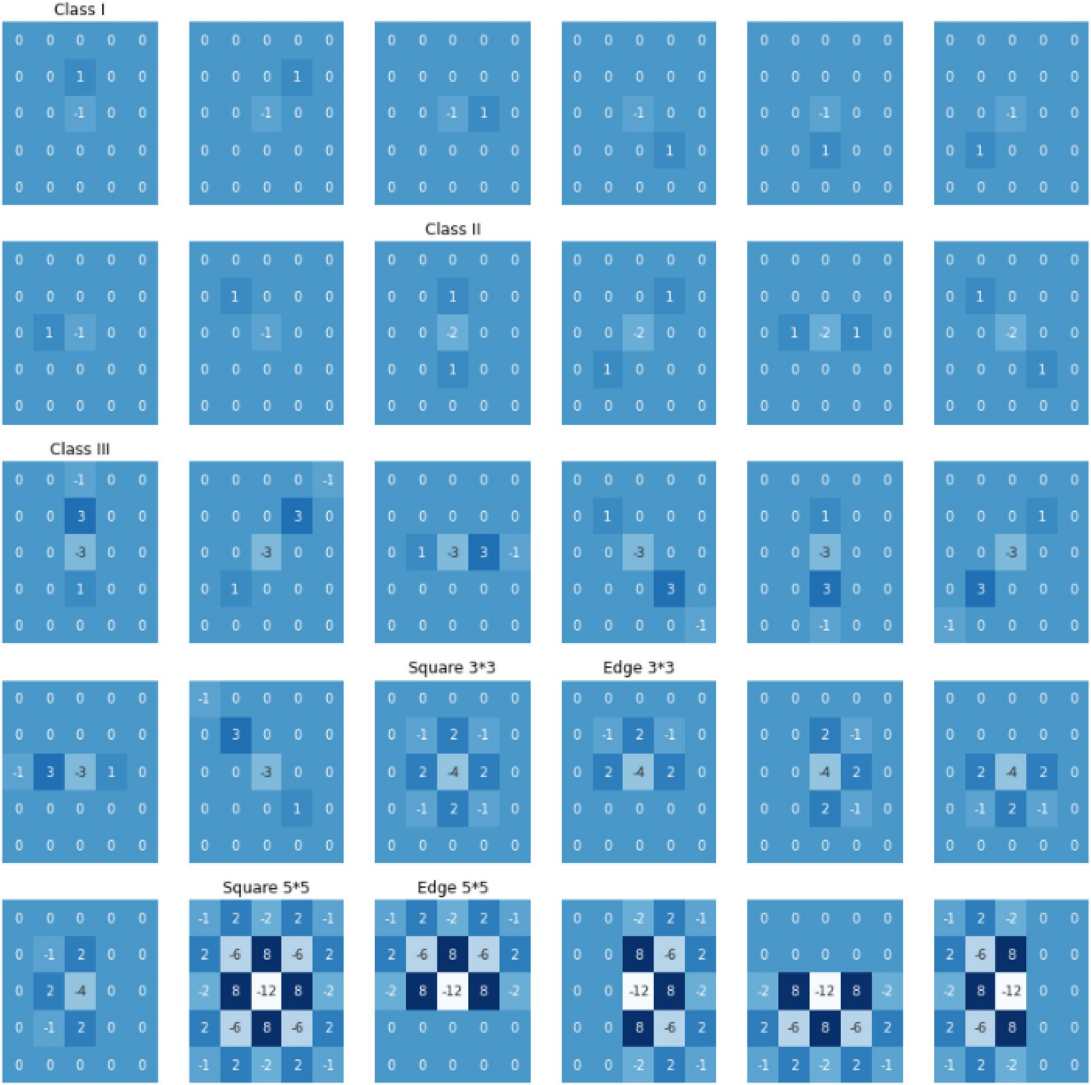
Set of 30 SRM Filters per category [10]

Next, SRM filters from YE-Net [31] are used, which are used to to pre-process our images [10]. These filters are very much capable of extracting useful features from our CXR images. There are 8 “Class I”, 4 “Class II” and 8 “Class III” filters. Moreover one 3*3 filter for square, 4 3*3 filters for edge, one filter with 5*5 dimensions for square and four filters of 5*5 for edge. All these filters are shown in Fig. 6. The filters which are not 5*5 are padded with 0s. In order to achieve better performance, the values of each filter is normalised in the range of [-1, 1]. Each value in the filter is divided by its maximum absolute value, i.e, 1 for “Class I”, 2 for “Class II”, 3 for “Class III”, 4 for “Square 3*3” and “Edge 3*3”, and 12 for “Square 5*5” and “Edge 5*5” [10].

### Performance metrics

We are using the following metrics to evaluate our proposed model along with other models - accuracy, loss, Area under ROC (AUC), trainable parameters, non-trainable parameters, model size, sensitivity, specificity, precision, F1-score.

1. Informally, accuracy is the fraction of predictions that our model has fulfilled. In binary context, accuracy is defined as follows:
  1 $$\begin{aligned} Accuracy = \frac{TN+TP}{TP+FN+FP+TN} \end{aligned}$$
2. The loss function is the categorical cross entropy loss and it is formally defined as follows:
  2 $$\begin{aligned} CE = -\sum _{i=i}^{C} t_{i}log(s_{i}) \end{aligned}$$
  In binary context, the equation is reduced to:
  3 $$\begin{aligned} CE = -t_{1}log(s_{1})-(1-t_{1})log(1-s_{1}) \end{aligned}$$
3. **AUC:** The overall performance is indicated by adding up all potential thresholds in the area below the ROC curve (AUC). For something like the ROC (receiver operating characteristic) curve, the algorithm performs better the if the curve is closer to the upper left corner and have greater the AUC.
4. **Sensitivity:** The percentage of samples that are actually positive and produce a positive result when employing the test in question is known as the test’s “Sensitivity” or “Recall” or “True Positive Rate” (TPR) (https://www.technologynetworks.com/analysis/articles/sensitivity-vs-specificity-318222). In our situation, it reveals the model’s sensitivity to COVID detection. It is defined formally as follows
  4 $$\begin{aligned} Sensitivity = \frac{TP}{FN+TP} \end{aligned}$$
5. **Specificity:** It measures the percentage of samples that are actually negative and yield a negative response with the test. It is also know as True Negative Rate (TNR) (https://www.technologynetworks.com/analysis/articles/sensitivity-vs-specificity-318222). In the case of our example, it explains how precisely it can determine whether a patient is not a COVID patient. It can be defined as follows:
  5 $$\begin{aligned} Specificity = \frac{TN}{FP+TN} \end{aligned}$$
6. The harmonic mean of the model’s precision and recall is known as the F1-score, which is given below
  6 $$\begin{aligned} F1-score = \frac{2*Recall*Precision}{Recall+Precision} \end{aligned}$$

### Experiment proceedings

**Fig. 7:**
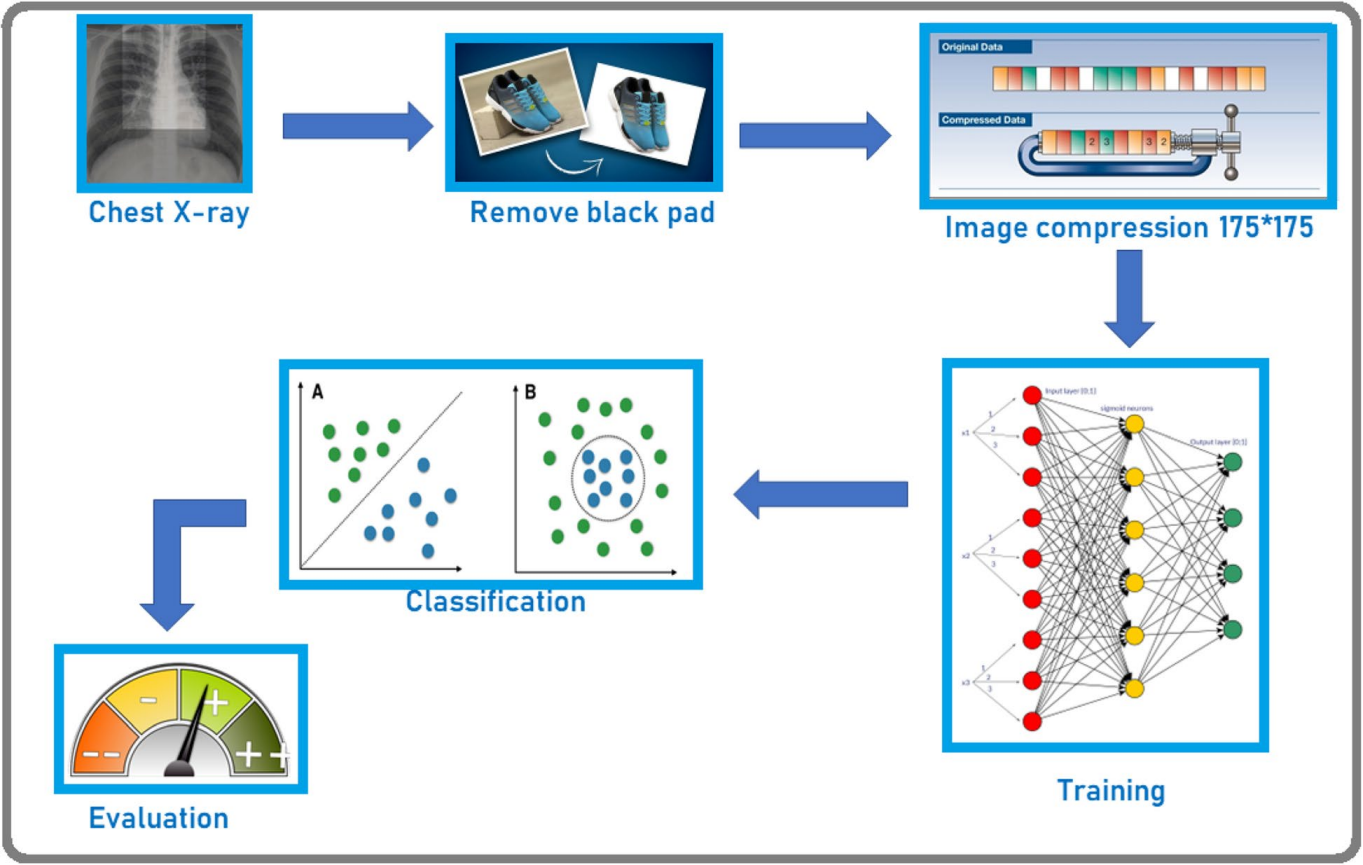
Experiment Setup

**Fig. 8:**
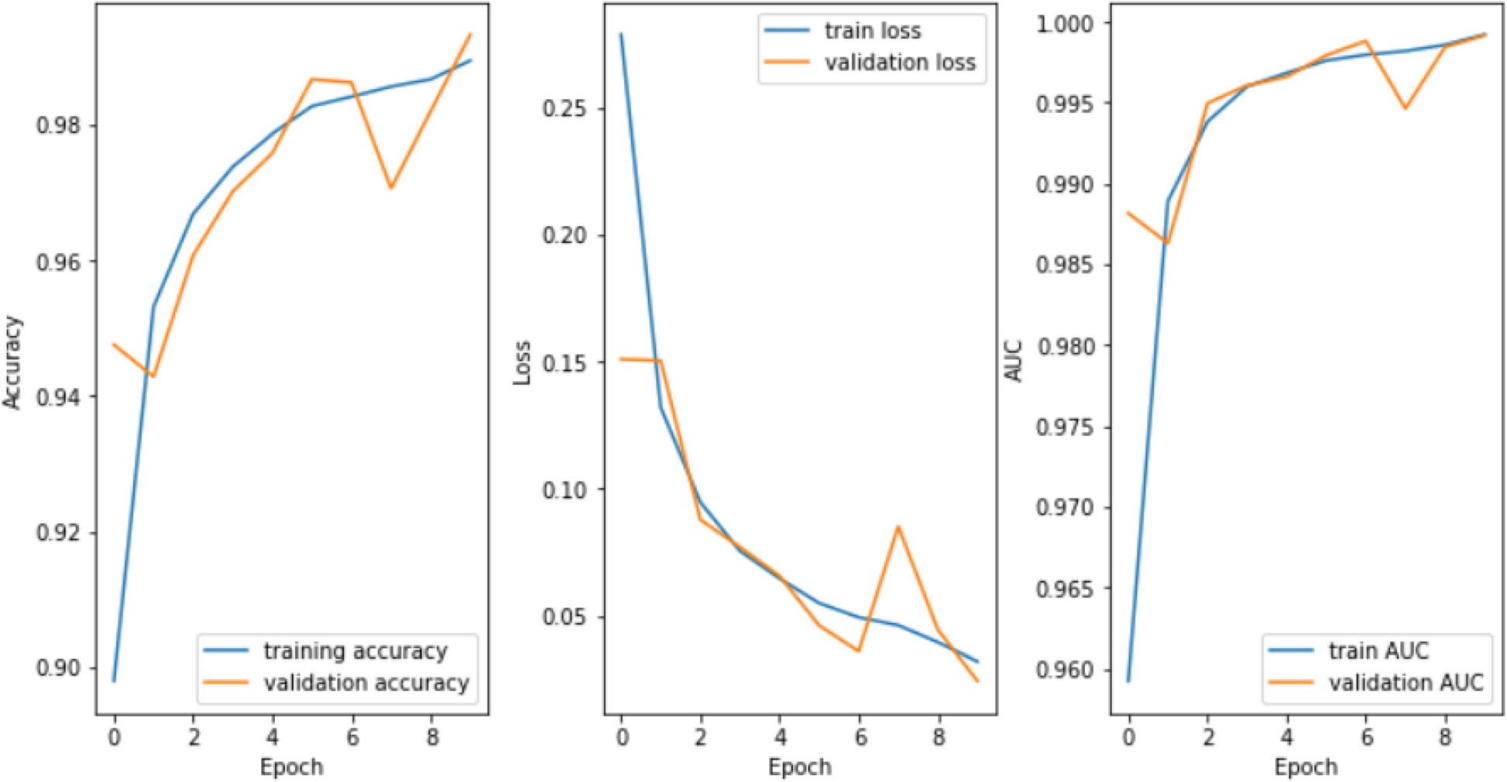
Graphs for accuracy, loss and AUC over 10 epochs

**Fig. 9:**
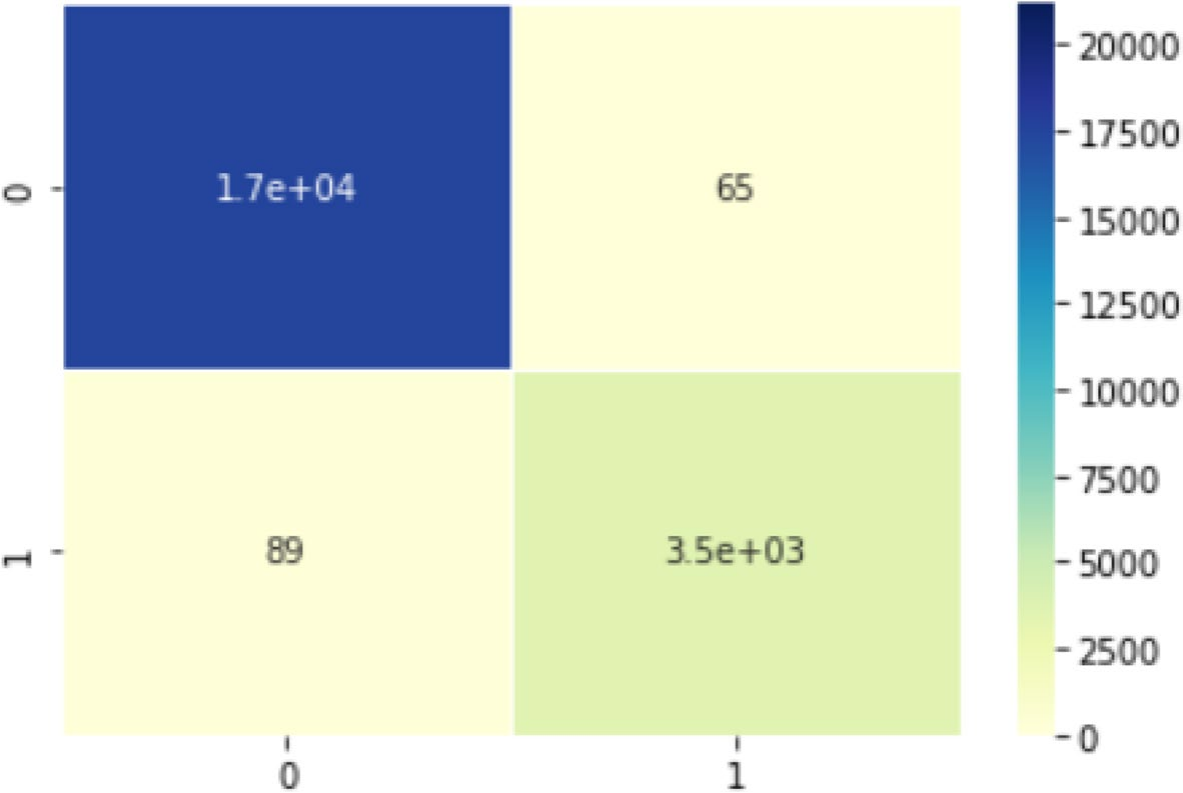
Confusion matrix of the proposed model

The experimental process is shown in Fig. 7: The pre-processed dataset is shuffled randomly and divided into 3 sets - training, test and validation datasets. These datasets are partitioned in such that 70% samples are training data, 10% samples are validation data and 20% samples are test data. All the models are trained and obtained accuracy, loss and AUC on training dataset. These performance plots are shown in Fig. 8. These trained models are experimented on test dataset and obtained test accuracy, test loss and test AUC. The testing performance in the form of confusion matrix is given in Fig. 9

Training process is carried out for 10 epochs with Adam optimiser and batch size of 33. The parameters of Adam optimiser is as follows: Learning Rate of 0.001, $\beta _{1}$ of 0.9, $\beta _{2}$ of 0.999, $\epsilon$ of $10^{-8}$ and no decay.

The proposed model, is comapared with 4 other existing models namely Deep GRU-CNN [19], CoroNet [13], CVDNet [3] and LMNet [18]. These results are shown in Table 2.

**Table 2:** Perforamcne measures of various models

	LMNet	CoroNet	Deep GRU-CNN	CVDNet	Proposed Model
Total Parameters	8,92,226	2,31,66,674	1,65,26,978	53,17,154	83,307
Trainable Parameters	8,92,162	2,31,12,146	8,23,490	53,15,618	81,647
Non-trainable parameters	64	54,528	1,57,03,488	1536	1660
Size in MB	10.89	278.36	69.55	64.13	1.18
Training Accuracy	95.69%	99.18%	97.4%	98.88%	99.47%
Training loss	0.1377	0.0286	0.0705	0.0296	0.0196
Training AUC	0.9893	0.9993	0.9973	0.9993	0.9995
Test Accuracy	92.27%	97.76%	93.48%	97.78%	98.91%
Test loss	0.2999	0.0719	0.1614	0.0713	0.0397
Test AUC	0.9684	0.9959	0.985	0.9962	0.9983
Sensitivity	0.7944	0.9545	0.9727	0.8939	0.9819
Specificity	0.9848	0.9819	0.9508	0.9969	0.9949
Precision	0.9289	0.9112	0.75	0.9851	0.9754
F1-score	0.8564	0.9323	0.847	0.9323	0.9786

From the table results it is observed that our proposed model generated a mere 83,307 parameters (better than others), out of which 81,647 are trainable while 1660 are non-trainable. The model size is only 1.18 MB (better than others). The model obtained a training loss of 0.0196 (better than others), training accuracy of 99.47% (better than others) and training AUC of 0.9995 (better than others). With test data, the model obtained loss of 0.0397 (better than others), accuracy of 98.91%(better than others) and AUC of 0.9983 (better than others). Out of 17,549 only 65 are mis-classified as COVID and out of 3616 only 89 are misclassified as normal. The sensitivity obtained is 0.9819 (better than others), the specificity obtained is 0.9949 (less than CVDNet), the precision is 0.9754 (less than CVDNet) and the F1-score is 0.9786 (better than others).

### Analysis

In case of LMNet,. the difference is high for metrics that are derived from training and test data. The accuracy differs by 3.42%. Even for metrics like loss and AUC, the difference is significant. So it is safe for us to conclude that LMNet has a tendency to overfit on our dataset. Similarly, Deep GRU-CNN have overfitting tendency. In Deep GRU-CNN case, the difference in accuracy is slightly more, about 3.92% (Refer Table 2).

In terms of training accuracy, the model performance is closest to CoroNet, which differs by merely 0.29%. But to reach to such a closeness, the model occupies a space of 278.36 MB (Refer Table 2). This is approximately 235.9 times more than our proposed model. So it is also safe for us to conclude that the tradeoff between accuracy and complexity of our proposed model is very low and it is possible for us to achieve both.

On comparing the difference between training accuracy and test accuracy, we get the following results: For Deep GRU-CNN, the difference is 3.92%, 3.42% for LMNet, 1.42% for CoroNet, 1.1% for CVDNet and only 0.56% in our proposed model (Refer Table 2). With this data, we can conclude that our model is less prone to overfitting on this dataset.

**Fig. 10:**
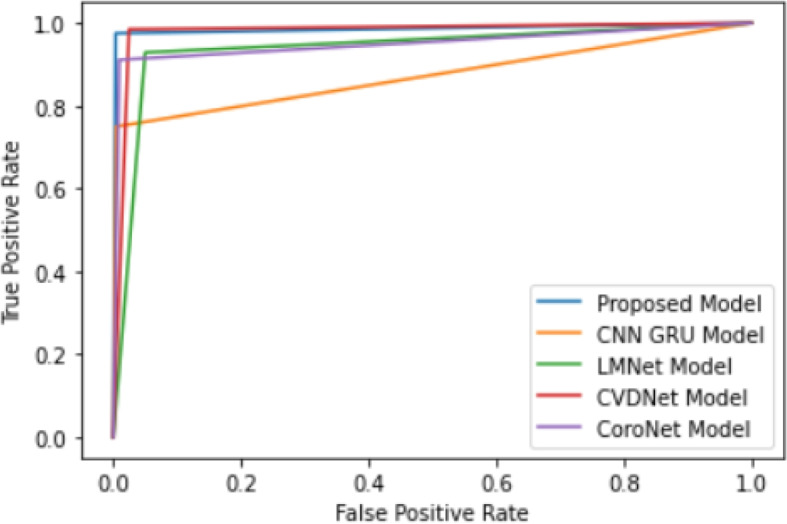
ROC Curve generated by all models

In the Fig. 10, our proposed model curve is upper left corner. The ROC curve clearly depicts the classification errors in a binary classification problem. So the ROC curve represents how effectively our model distinguishes between the positive class and the negative class. While other models are negligibly away from our model, the Deep GRU-CNN deviates a lot from the results of our proposed model. So we can safely conclude from the graph that our proposed model effectively classifies any instance present in our dataset.

Further, the proposed model is requires less number of trainable parameters hence can be easily deployed in any edge devices.

## Conclusion

A novel CNN based lightweight model for COVID-19 detection from CXR scans is proposed in this paper. The model has 83,307 parameters, 81,647 of which are trainable and 1660 of which are not. The dataset used consisted of 21,165 CXR images with each image of 299*299 pixel. The images are divided into four categories: normal, lung opacity, viral pneumonia, and COVID-19. The dark padding surrounded the CXR photos is removed, and the images are resized to 175*175 pixels. The complete dataset was divided into training, validation and test datasets in 7:1:2 ratio respectively. On test dataset our proposed light weight deep learning model achieved 99.47% accuracy and 97.86% F1-score. When compared with other state-of-the-art models the proposed lightweight model outperformed in terms of accuracy and F1-score. As the proposed model requires few trainable parameters it can easily deployed in any edge device for COVID-19 detection.

## Data Availability

The datasets used in our research are public available. The datasets generated and/or analysed during the current study are available in following web links: https://bimcv.cipf.es/bimcv-projects/bimcv-covid19/#1590858128006 9e640421-6711https://github.com/ml-workgroup/covid-19-image-repository/tree/master/pnghttps://sirm.org/category/senza-categoria/covid-19/https://eurorad.orghttps://github.com/ieee8023/covid-chestxray-datasethttps://figshare.com/articles/COVID-19 Chest X-Ray Image Repository/12580328https://github.com/armiro/COVID-CXNethttps://www.kaggle.com/c/rsna-pneumonia-detection-challenge/datahttps://www.kaggle.com/paultimothymooney/chest-xray-pneumonia.
